# Genome-Wide Identification and Expression Analysis of Sucrose Nonfermenting 1-Related Protein Kinase (*SnRK*) Genes in *Salvia miltiorrhiza* in Response to Hormone

**DOI:** 10.3390/plants13070994

**Published:** 2024-03-30

**Authors:** Tingyao Liu, Yinkai Yang, Ruiyan Zhu, Qichao Wang, Yao Wang, Min Shi, Guoyin Kai

**Affiliations:** 1 College of Horticulture, Shenyang Agricultural University, Shenyang 110866, China; 2 Zhejiang Provincial TCM Key Laboratory of Chinese Medicine Resource Innovation and Transformation, Zhejiang International Science and Technology Cooperation Base for Active Ingredients of Medicinal and Edible Plants and Health, Jinhua Academy, School of Pharmaceutical Sciences, Academy of Chinese Medical Sciences, Zhejiang Chinese Medical University, Hangzhou 310053, China; 3 State Key Laboratory of Phytochemistry and Plant Resources in West China, Kunming Institute of Botany, Chinese Academy of Sciences, Kunming 650201, China

**Keywords:** *S. miltiorrhiza*, SnRK family, bioinformatics analysis, expression pattern, phytohormone

## Abstract

The *SnRK* gene family is the chief component of plant stress resistance and metabolism through activating the phosphorylation of downstream proteins. *S. miltiorrhiza* is widely used for the treatment of cardiovascular diseases in Asian countries. However, information about the *SnRK* gene family of *S. miltiorrhiza* is not clear. The aim of this study is to comprehensively analyze the *SnRK* gene family of *S. miltiorrhiza* and its response to phytohormone. Here, 33 *SmSnRK* genes were identified and divided into three subfamilies (SmSnRK1, SmSnRK2 and SmSnRK3) according to phylogenetic analysis and domain. *SmSnRK* genes within same subgroup shared similar protein motif composition and were unevenly distributed on eight chromosomes of *S. miltiorrhiza*. *Cis*-acting element analysis showed that the promoter of *SmSnRK* genes was enriched with *ABRE* motifs. Expression pattern analysis revealed that *SmSnRK* genes were preferentially expressed in leaves and roots. Most *SmSnRK* genes were induced by ABA and MeJA treatment. Correlation analysis showed that *SmSnRK3.15* and *SmSnRK3.18* might positively regulate tanshinone biosynthesis; *SmSnRK3.10 and SmSnRK3.12* might positively regulate salvianolic acid biosynthesis. RNAi-based silencing of *SmSnRK2.6* down-regulated the biosynthesis of tanshinones and biosynthetic genes expression. An in vitro phosphorylation assay verified that SmSnRK2.2 interacted with and phosphorylated SmAREB1. These findings will provide a valuable basis for the functional characterization of *SmSnRK* genes and quality improvement of *S. miltiorrhiza.*

## 1. Introduction

Plants have developed a complicated and multilayer system to recognize and react to abiotic stresses, including drought, salinity and extreme temperature [1]. The post-translational modification of proteins, especially the phosphorylation and dephosphorylation process, is vital for plants to deal with different stresses [2]. The sucrose non-fermenting 1-related protein kinases (SnRK) are Ser/Thr protein kinases, which phosphorylate downstream target proteins for the transduction of signal pathways [3]. Based on sequence similarity and protein structure, SnRK could be subdivided into three subfamilies in plants: SnRK1, SnRK2 and SnRK3 [4]. Due to the Ser/Thr kinase domain located on the N-terminal of SnRKs, the similarity of the N-terminal was higher than the C-terminal. The function of three subfamilies was not independent and the interaction of these genes forms a large and sophisticated regulation network [5].

The plant SnRK1, heterotrimeric complexes consisting of three subunits (a catalytic α-subunit and regulatory *β*- and *γ*- subunits), is an ortholog of the sucrose nonfermenting 1 (SNF1) of yeast and AMP-activated protein kinase (AMPK) of mammals [6]. Plant SnRK1 contains a protein kinase domain, ubiquitin-associated domain (UBA) and kinase-associated 1 domain (KA1) [7]. The SnRK1 family is a relatively small subfamily, containing three members in *Arabidopsis thaliana* and four members in *Oryza sativa*. SnRK1 kinase is a central regulator of energy management and drives a large array of metabolic and transcriptional reprogramming in the response of metabolic signaling and stress. SnRK1 is rapidly activated by stress and energy deficiency, thereby phosphorylating metabolic enzymes or bZIP transcription factors (TFs), leading metabolism-related gene activation or depression. Moreover, the function of SnRK1 is not limited to stress responses, and many studies have shown that SnRK1 balances metabolism, growth and development [8,9,10].

The SnRK2 subfamily is a plant-specific gene family. Both *A. thaliana* and *O. sativa* have 10 members in this subfamily. Most of SnRK2s in *A. thaliana* and *O. sativa* are activated by ABA and osmotic stress [11,12]. SnRK2s contain a Pkinase domain at the N-terminal and a dissimilar regulatory domain at the C-terminal [13]. SnRK2s play an important role when plants are confronted with abiotic stresses and they are central kinases in ABA signaling [14]. ABA could release the SnRK2s from the suppression of PP2Cs. These activated SnRK2s phosphorylate target proteins [15,16,17], such as SLAC1 [18], KAT1 [19], AtRbohF [20] and TFs [14,15]. SnRK2.2, SnRK2.3 and SnRK2.6 are central and positive regulators of ABA signaling in *A. thaliana*. The triple mutant of *snrk2.2*, *snrk2.3* and *snrk2.6* is extremely insensitive to the ABA-mediated inhibition of seed germination [21]. Studies have also shown that SnRK2s could enhance tolerance to salinity and drought in plants [22,23].

The SnRK3 subfamily is also plant-specific, and is also known as CIPK (CBL-interacting protein kinase). Calcineurin B-like proteins (CBLs) are calcium sensors, interacting with SnRK3 to transfer cellular calcium signals. *A. thaliana* and *O. sativa* have 26 and 33 members in this subfamily [24,25]. SnRK3 contains a Pkinase domain at the N-terminal and an NAF domain at the C-terminal. When plants are confronted with stresses, CBLs detect and bind to Ca^2+^ ions, interacting with the NAF domain of SnRK3s to activate it [26,27]. The CBL-CIPK module plays an important role in the salt stress of plants. The first discovered CBL-CIPK module was the SOS (salt overly sensitive) module. In *A. thaliana*, the cell membrane located SOS3 (AtCBL4) recognized and bound to a calcium signal that was produced by salt stress. Then, SOS3 formed a complex with SOS2 (AtCIPK24), which activated the AtCIPK24 kinase activity to phosphorylate SOS1 (Na^+^/H^+^ antiporter) to excrete Na^+^ from plant cells and protect plant cells from the damage caused by a high concentration of Na^+^ [28,29,30]. Moreover, CBL-CIPK modules are also involved in ABA signaling. The CBL1 and CIPK15 modules negatively regulated seed germination, stomatal movement and ABA-mediated gene expression [31]. CIPK1 and CIPK23 also played a negative role in ABA-regulated seed germination and stomatal movement, respectively [32,33]. CIPK11 phosphorylated and activated ABI5 (ABA-Insensitive 5) to regulate seed germination [34].

The traditional Chinese medicinal plant *S. miltiorrhiza* (also known as Danshen) is widely used alone or in combination with other medicines in clinics for the management of cardiovascular diseases in China and even in some other Asian countries [35]. Many drugs containing *S. miltiorrhiza* material have been developed and marketed in China for preventing and treating cardiovascular diseases, such as Fufang Danshen tablets and Compound Danshen Dripping Pills [36]. With the genome of *S. miltiorrhiza* sequenced [37,38,39], many gene families have been identified and investigated in *S. miltiorrhiza*, such as WRKY [40], bHLH [41], TIFY [42], laccases [43], B-box [44] and MADS-box [45]. The information about *SnRK* genes in *S. miltiorrhiza* is not clear despite *SnRK* genes being key components of energy management and stress resistance in plants. Moreover, the function of *SnRK* genes in *S. miltiorrhiza* is also obscure. To better understand the information and function of *SnRK* genes in *S. miltiorrhiza*, we systematically analyzed *SmSnRK* genes with bioinformatics.

In this study, we identified thirty-three *SnRK* gene family members in *S. miltiorrhiza*, including three members in SnRK1, seven members in SnRK2 and twenty-three members in SnRK3. Tissue expression pattern, gene structure, phylogenetic relationships, hormone response and chromosome location were systematically analyzed. Based on correlation and function analysis, we found that SmSnRK2.6 positively regulated tanshinones biosynthesis and gene expression. These results will provide important insights into the molecular mechanisms of SnRK for stress tolerance and molecular breeding in *S. miltiorrhiza*.

## 2. Results

### 2.1. Identification, Phylogenetic Analysis, Motif and Chromosomal Location of SnRK Genes in S. miltiorrhiza

A total of 33 *SmSnRK* genes were identified in the genome of *S. miltiorrhiza* with BLAST search and HMMscan, and the longest protein sequence was selected for further study. All of the 33 *SmSnRK* genes possessed a Ser/Thr kinase domain (Pkinase, PF00069) and can be divided into three subfamilies (SmSnRK1, SmSnRK2 and SmSnRK3) based on different domains contained in these genes. *SmSnRK1.1*, *SmSnRK1.2* and *SmSnRK1.3* were placed into the SnRK1 subfamily, due to these genes containing the Pkinase domain (PF00069), UBA domain (PF00627) and KA1 domain (PF02149) domain. *SmSnRK2.1*–*SmSnRK2.7* were identified as part of the SnRK2 subfamily, as these seven members showed high similarity with the SnRK2 family in *A. thaliana* and *O. sativa*. The 23 members (*SmSnRK3.1*–*SmSnRK3.23*) grouped into SnRK3 subfamily had an NAF domain (PF03822). The residues of the amino acids of the *SmSnRK* genes ranged from 341 (*SmSnRK2.1*) to 513 (*SmSnRK1.1*) and the molecular weight of the *SmSnRK* genes ranged from 38.78 to 58.52 kDa. The isoelectric point of the *SmSnRK* gene family spanned from 4.838 to 9.633. Based on subcellular localization prediction results, most of the SmSnRK proteins were located in the cytoplasm and nucleus, followed by the chloroplast (Table S1).

**Figure 1:**
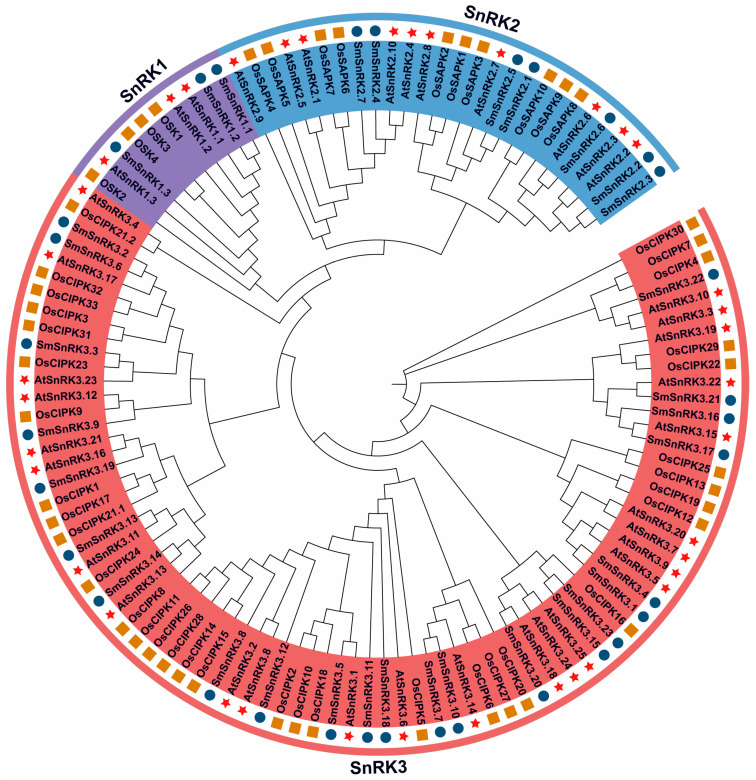
Phylogenetic tree of *SnRK* genes from *S. miltiorrhiza*, *A. thaliana* and *O. sativa*. A total of 33 *SmSnRK* genes, 38 *AtSnRK* genes and 48 *OsSnRK* genes were clustered into three subgroups. Background purple, blue, and red areas represent SnRK subfamilies 1, 2 and 3, respectively. The stars, circle and squares represent the SnRK from *A. thaliana*, *S. miltiorrhiza* and *O. sativa*, respectively. The tree was built by MEGA7 with the neighbor-joining method.

To further study the phylogenetic relationships of *SmSnRK* genes, we constructed a phylogenetic tree using the Neighbor-Joining (NJ) method with the full-length amino acid sequences of the *SnRKs* genes from *S. miltiorrhiza*, *A. thaliana* and *O. sativa*. As shown in Figure 1, all *SnRKs* genes were clustered into three groups, which was basically in accordance with previous studies. *SmSnRK* genes were more related to the *SnRKs* genes of *A. thaliana* than *O. sativa*. In detail, *SmSnRK2.2*, *SmSnRK2.3* and *SmSnRK2.6* had a close relationship with *AtSnRK2.2*, *AtSnRK2.3* and *SmSnRK2.6*, respectively, but not *OsSAPK8*. *SmSnRK1.1* and *SmSnRK1.2* were clustered with *AtSnRK1.1*.

Twelve conserved motifs were identified in SmSnRK proteins and all SmSnRK proteins had the conserved Pkinase domain, including motif 1, 2, 3, 4, 5 and 7. Moreover, SmSnRK proteins within the same subfamily retained a similar composition pattern. Motif 2, 6 and 9 were the NAF domain, specifically found in the SmSnRK3 subfamily members. Motif 14 (UBA domain) and 15 (KA1 domain) were specifically contained in SmSnRK1 subfamily members (Figure S1a). The conserved motif compositions supported the subfamily classifications.

Gene structural analysis showed that the members in the subgroup shared a similar distribution of exon numbers. All of the *SmSnRK1* members have ten exons. All *SmSnRK2* members have nine exons, except for *SmSnRK2.2* and *SmSnRK2.3* which both clustered in a subgroup and contained eight exons (Figure S1b).

A chromosomal distribution map of *SmSnRK* genes was established and it was found that *SmSnRK* genes were distributed randomly on eight chromosomes. In addition, only the *SmSnRK3.15* gene was located on chromosome LG2, whereas most of the *SmSnRK* genes (eight genes) were located on chromosome LG8 (Figure S2).

### 2.2. Analysis of Cis-Elements in the Promoter of SmSnRK Genes

**Figure 2:**
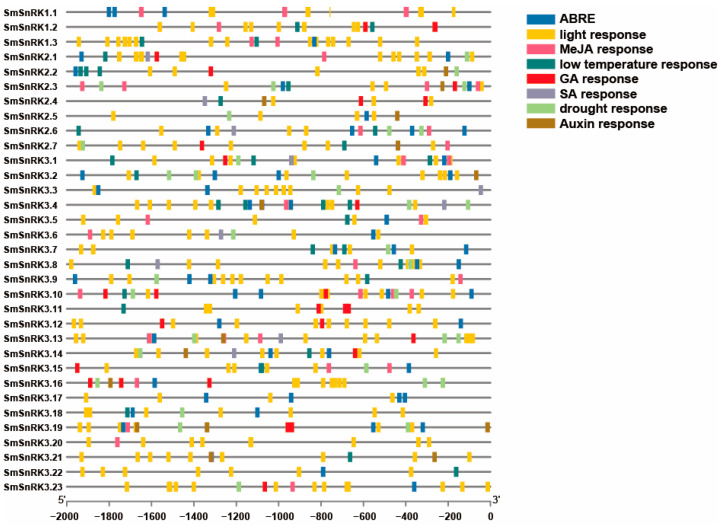
Analysis of *cis*-elements in the promoter regions of *SmSnRK* genes. Different motifs were represented with rectangles of different colors as seen in the figure legend, top left.

To understand the potential biological regulatory mechanisms of the *SmSnRK* genes, the promoter sequence (2 kb region upstream of ATG) was analyzed with PlantCARE. The *cis*-elements in the promoter of *SmSnRK* genes can be classified into light response, stress response (low temperature stress and drought stress) and hormone response (ABA, JA, Auxin, GA and SA). Except for *SmSnRK3.20*, the promoter of all *SmSnRK* genes contained an *ABRE cis*-element, indicating that most *SmSnRK* genes may be involved in ABA signaling. Moreover, about 67%, 51.5%, 36% and 30% of the promoters of *SmSnRK* genes contained MeJA, GA, auxin and SA responsive motifs, respectively. The drought response *cis*-elements were enriched in the *SmSnRK2* and *SmSnRK3* subfamily, but not in *SmSnRK1* (Figure 2). The promoter analysis showed that most of the *SmSnRK* genes may participate in diverse hormone responses and drought stress. These results suggested that different subfamilies may be regulated by distinct pathways.

### 2.3. Expression Patterns of SmSnRK Genes

**Figure 3:**
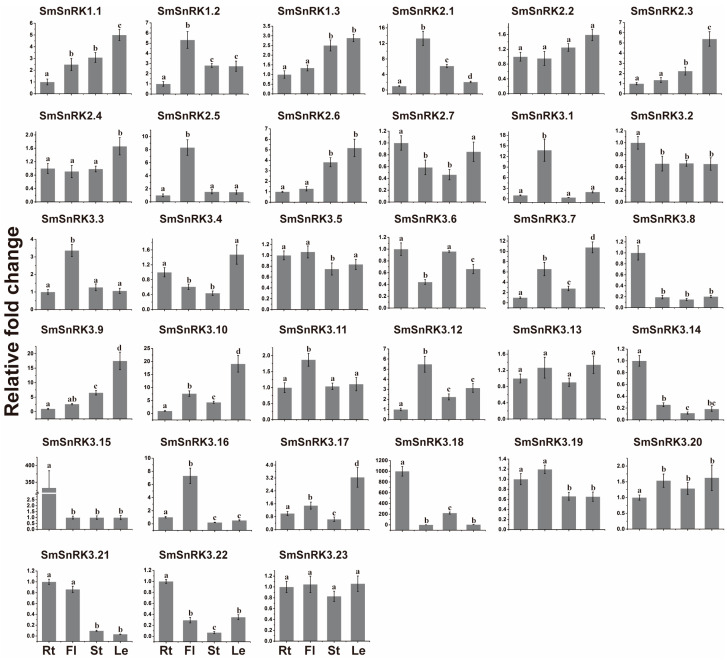
Expression patterns of *SmSnRK* genes in different tissues of *S. miltiorrhiza*. Roots, stems, leaves and flowers are represented as Rt, St, Le and Fl, respectively. *SmActin* gene was used as the reference gene. Error bars represent standard deviations of mean value from three biological and three technical replicates. Different letters (a, b, c and d) represent different significance, *p* < 0.05.

To gain insight into the relative expression level of *SmSnRK* genes in different tissues, the flower, leaf, stem and root were sampled for qRT-PCR. *SmSnRK* genes exhibited differential expression in different tissues, even in the same subgroup. *SmSnRK1.1* and *SmSnRK1.3* showed a similar expression pattern with the main expression in leaves, followed by stem, flower and root. Most of *SmSnRK2* subfamily members showed high expression in leaves, including *SmSnRK2.2*, *SmSnRK2.3*, *SmSnRK2.4* and *SmSnRK2.6*. *SmSnRK2.1* and *SmSnRK2.5* were mainly expressed in flowers. The expression pattern of *SmSnRK3s* could be grouped into three categories. *SmSnRK3.1*, *SmSnRK3.3*, *SmSnRK3.11*, *SmSnRK3.12* and *SmSnRK3.16* showed high expression in flowers. *SmSnRK3.2*, *SmSnRK3.8*, *SmSnRK3.14*, *SmSnRK3.15*, *SmSnRK3.18* and *SmSnRK3.22* were mainly expressed in roots. *SmSnRK3.13*, *SmSnRK3.20* and *SmSnRK3.23* showed relatively equivalent expression levels in the tested tissues. The diverse expression levels of *SmSnRKs* in different tissues suggested that *SmSnRKs* exhibit functional diversity in plant development and stress response (Figure 3).

**Figure 4:**
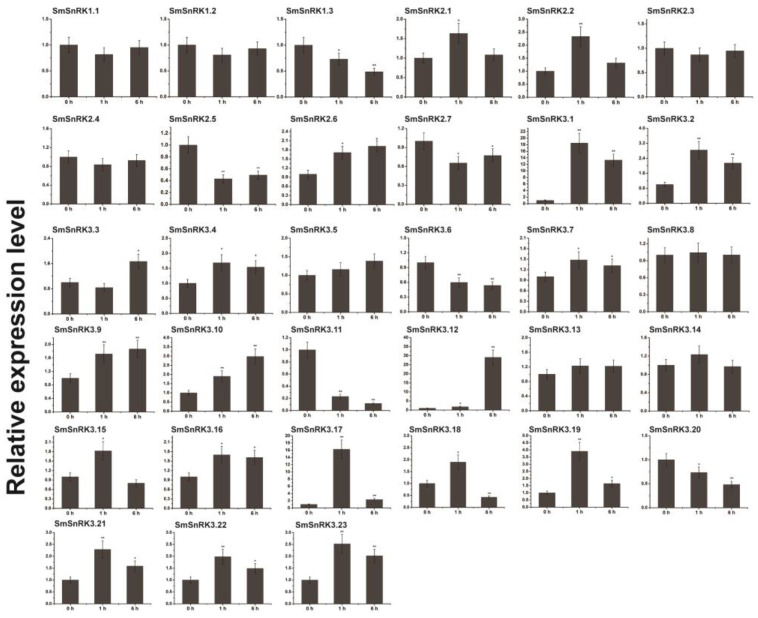
Expression patterns of *SmSnRK* genes with ABA treatment. The hairy roots treated with 200 μM ABA for 0, 1 and 6 h. *SmActin* gene was used as the reference gene. Error bars represent standard deviations of mean value from three biological and three technical replicates. Statistical significance was determined based on Student’s *t*-test (* *p* < 0.05 and ** *p* < 0.01).

**Figure 5:**
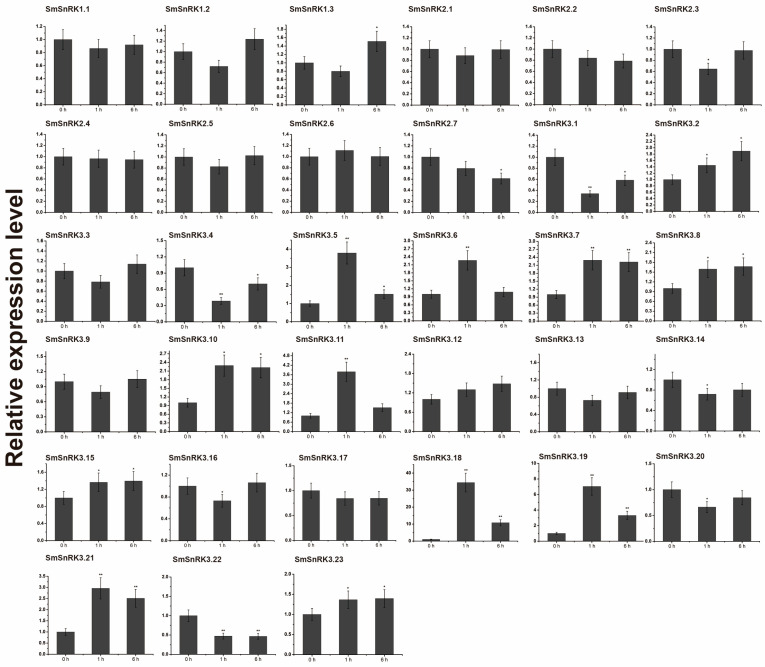
Expression patterns of *SmSnRK* genes with MeJA treatment. The hairy roots treated with 100 μM MeJA for 0, 1 and 6 h. *SmActin* gene was used as the reference gene. Error bars represent standard deviations of mean value from three biological and three technical replicates. Statistical significance was determined based on Student’s *t*-test (* *p* < 0.05 and ** *p* < 0.01).

It was reported that MeJA (methyl jasmonate) [46] and ABA (abscisic acid) [47] were important hormones in promoting tanshinone and salvianolic acid accumulation. Moreover, the promoter of *SmSnRK* genes was enriched with ABA-responsive and MeJA-responsive *cis*-elements [48]. To determine whether MeJA and ABA affect the expression of *SmSnRK* genes, we treated *Salvia miltiorrhiza* with ABA and MeJA. Quantitative real-time PCR (qRT-PCR) showed that most of the *SmSnRK* genes (45%) were up-regulated by ABA treatment and several members (24%) were down-regulated by ABA treatment. The expression of *SmSnRK3.1*, *SmSnRK3.12* and *SmSnRK3.17* was highly induced by ABA (Figure 4). One-third of *SmSnRK* gene expression was induced by MeJA, of which *SmSnRK3.18* and *SmSnRK3.19* were extremely induced. A limited number of *SmSnRK* members were down-regulated by MeJA treatment (Figure 5). The expression of *SmSnRK3.2*, *SmSnRK3.10*, *SmSnRK3.18*, *SmSnRK3.19* and *SmSnRK3.21* was responsive to both ABA and MeJA. These results suggested that the expression of *SmSnRKs* in different tissues and the response to hormones were varied, even in the same subgroups.

### 2.4. Gene Ontology Analysis of SmSnRKs

**Figure 6:**
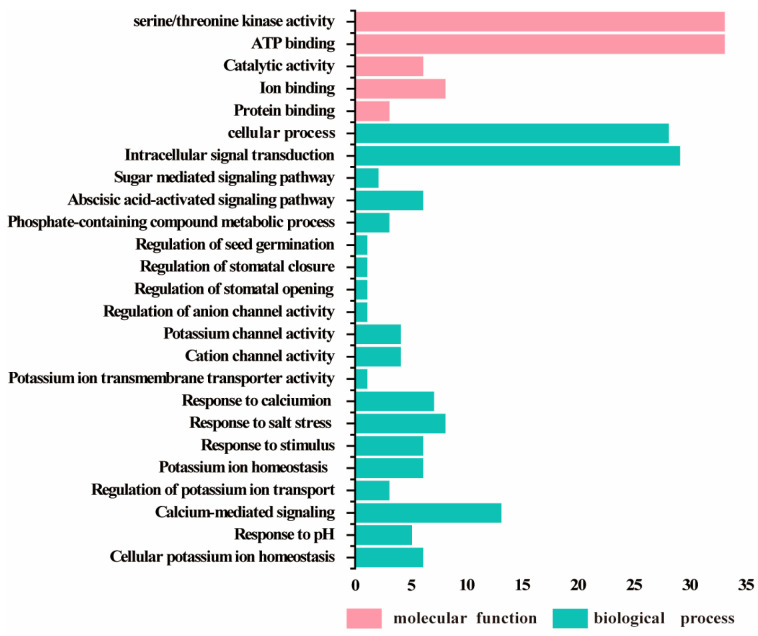
Gene ontology of SmSnRK2 proteins.

Gene ontology analysis revealed that the molecular function of all of the *SmSnRK* genes was closely related to ATP binding and protein serine/threonine kinase activity, followed by protein binding, catalytic activity and ion binding. The biological processes of most *SmSnRK* genes were associated with cell progress and intracellular signal transduction, indicating that *SmSnRK* genes played an important role in the signaling pathway. Moreover, several *SmSnRK* members were involved in ABA signaling, calcium-mediated signaling and calcium/potassium homeostasis (Figure 6).

### 2.5. Correlation Analysis of SmSnRK Genes with Tanshinones and Salvianolic Acids Biosynthesis Genes

**Figure 7:**
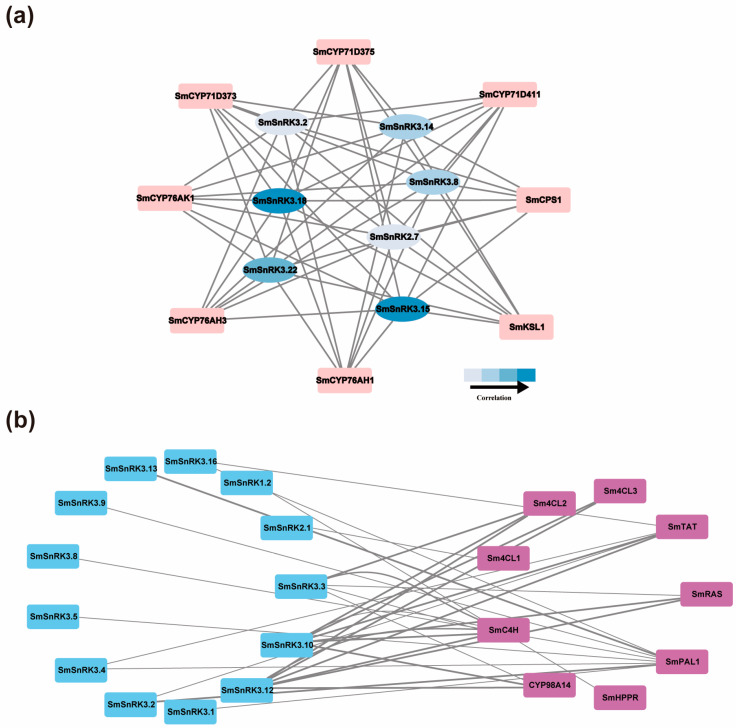
(**a**) Correlation analysis of *SmSnRK* genes with tanshinone biosynthesis genes. Pearson’s correlation coefficients of gene pairs > 0.8 and *p* < 0.01 were considered significant. The different correlation levels of the gene pairs are marked by different colors (White: low correlation; Blue: high correlation). (**b**) Correlation analysis of *SmSnRK* genes with salvianolic acid biosynthesis genes. Pearson’s correlation coefficients of gene pairs > 0.8 and *p* < 0.01 were considered significant. The different correlation levels of the gene pairs are marked by different width of lines (Narrow: low correlation; Thick: high correlation).

To further study whether *SmSnRK* genes had potential regulatory roles in the expression of tanshinone biosynthesis genes, we employed a co-expression analysis of *SmSnRK* genes and tanshinone biosynthesis genes, based on the tissue expression patterns and MeJA transcriptome (Figure S3). *SmSnRK3.15* and *SmSnRK3.18* were relatively highly co-expressed with key tanshinone biosynthesis genes. *SmSnRK3.8*, *SmSnRK3.14* and *SmSnRK3.22* showed moderate co-expression with key tanshinone biosynthesis genes, suggesting that *SmSnRK* genes could be participating in tanshinone accumulation (Figure 7a). Moreover, we also used the ABA transcriptome-based (Figure S4) correlation analysis to explore the relationship between *SmSnRK* genes and salvianolic acids biosynthesis genes. It was observed that *SmSnRK3.10* and *SmSnRK3.12* had strong positive correlations with all of the salvianolic acids biosynthesis genes, except *SmPAL1* and *SmHPPR*, suggesting that these two *SmSnRK* genes might regulate the salvianolic acid biosynthesis genes expression (Figure 7b).

### 2.6. SmSnRK2.6 Positively Regulated Tanshinones Biosynthesis and Genes Expression

**Figure 8:**
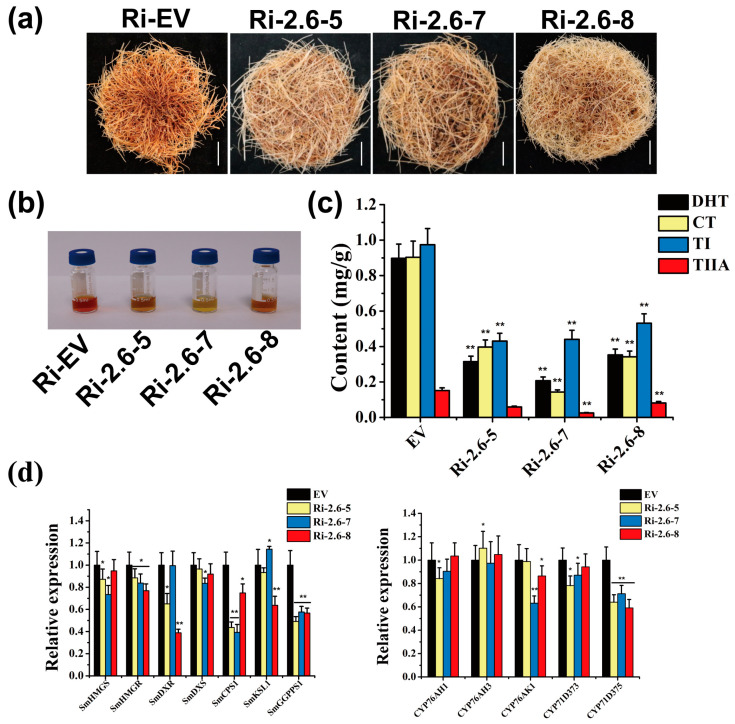
SmSnRK2.6 is a positive regulator of tanshinone biosynthesis. (**a**) The phenotypes of hairy roots (Ri-EV and Ri-SmSnRK2.6 hairy root lines) pictured after 45 d cultured in 1/2 MS liquid medium. Scale bar, 1 cm. (**b**) Representative images showing the extracts of tanshinones of (**a**). (**c**) Determination of CT, TIIA, DHT, and TI in the hairy roots (Ri-EV and Ri-SmSnRK2.6 hairy root lines). (**d**) Relative expression of tanshinone biosynthesis structural genes in Ri-EV and Ri-SmSnRK2.6 lines. *Actin* was used as an internal reference gene. All data represent the means ± SD of three biological replicates. Statistical significance was determined based on Student’s *t*-test (* *p* < 0.05 and ** *p* < 0.01).

Previous studies have demonstrated that SmSnRK2.6 regulates the accumulation of phenolic acids and is involved in the ABA signaling and stress response in *S. miltiorrhiza* [49]. Moreover, based on our correlation analysis, we found that SmSnRK2.6 was related to tanshinones biosynthesis, indicating that SmSnRK2.6 may participate in tanshinones biosynthesis. To investigate whether SmSnRK2.6 participated in regulating tanshinone biosynthesis, we silenced SmSnRK2.6 expression in transgenic hairy root lines with the RNAi method. Ri-2.6-5, Ri-2.6-7 and Ri-2.6-8 with substantial a down-regulation of SmSnRK2.6 expression were selected for further study (Figure S5). The phenotype and extract color of hairy roots hinted that the content of tanshinones in Ri-SmSnRK2.6 strains was lower than that in the control strain (Figure 8a,b). Metabolic content analysis showed that the content of dihydrotanshinone (DHT), cryptotanshinone (CT), tanshinone I (TI) and tanshinone IIA (TIIA) in the control lines was about 2.54–4.33, 2.28–6.29, 1.83–2.26 and 1.83–2.26 times higher than in the Ri-SmSnRK2.6 lines, respectively (Figure 8c). Moreover, the expression level of most tanshinone biosynthetic genes was decreased in the Ri-SmSnRK2.6 lines, among which SmGGPPS1 and SmCPS1 were significantly down-regulated, with the lowest decrease of 0.26- and 0.43-fold that of the control lines, respectively (Figure 8d). These results confirmed that SmSnRK2.6 positively regulated tanshinone biosynthesis.

### 2.7. SmSnRK2.2 Interacts with and Phosphorylates SmAREB1

**Figure 9:**
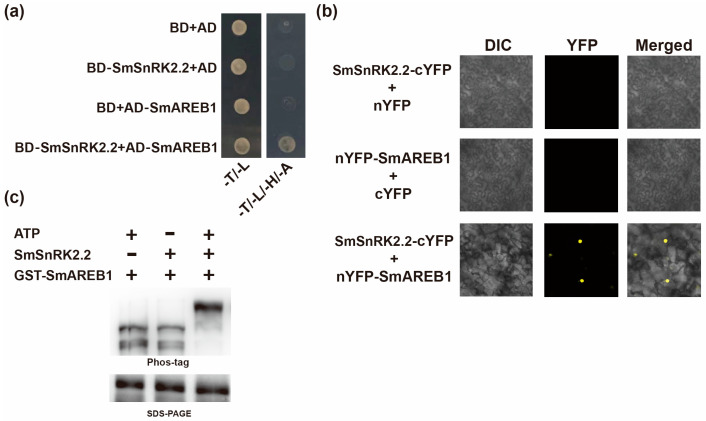
(**a**) Y2H assay to detect interactions between SmSnRK2.2 and SmAREB1 proteins. -T/-L, dropout medium lacking Trp and Leu. -T/-L/-H/-A, dropout medium lacking Trp, Leu, His and Ade. (**b**) BiFc analyses to detect interactions between SmSnRK2.2 and SmAREB1 proteins in tobacco leaves. SmAREB1 was fused to the N-terminal fragment of YFP and SmSnRK2.2 was fused to the C-terminal fragment. (**c**) In vitro phosphorylation assays to detect the phosphorylation of SmAREB1 by SmSnRK2.2. The mobility shift of SmAREB1 was detected using immunoblot analyses with anti-GST antibody.

A previous study has shown that SmSnRK2.3 and SmSnRK2.6 interacted with SmAREB1 [49]. To test whether SmSnRK2.2 interacts with SmAREB1, yeast two hybrid (Y2H) assays and bimolecular fluorescence complementation (BiFc) assays were applied. Y2H assays showed that SmSnRK2.2 interacts with SmAREB1 in yeast (Figure 9a). BiFc assays showed that SmSnRK2.2 can interact with SmAREB1 in plant cells (Figure 9b). Furthermore, we also tested whether SmSnRK2.2 could phosphorylate SmAREB1 by using a Phos-tag reagent. As shown in Figure 9c, the mobility shift was quite slow compared with the control, indicating that SmSnRK2.2 could phosphorylate SmAREB1 in vitro.

## 3. Discussion

An increasing number of studies have revealed that the SnRK family is a crucial component in plant signaling transduction and response to abiotic stress [50,51]. Moreover, the SnRK family members of many plants have been clarified, including *Liriodendron chinense* [52], *Brassica napus* [53], *Casuarina equisetifolia* [54], *Brachypodium distachyon* [55], cotton [56], *Triticum aestivum* [57] and *Phaseolus vulgaris* [58]. However, only a few *SnRK* genes of *S. miltiorrhiza* were cloned and studied in previous studies [59]. In this study, we systematically identified a total of thirty-three *SmSnRK* members in the genome of *S. miltiorrhiza*, including three *SmSnRK1* genes, seven *SmSnRK2* genes and twenty-three *SmSnRK3* genes. The component ratio of *SmSnRK* subfamily genes in all *SmSnRK* genes were similar to other species, in which *SmSnRK1* subfamily genes made up a small percentage of *SmSnRK* genes and *SmSnRK3* subfamily genes accounted for a large proportion of *SmSnRK* genes.

It was reported that *SmSnRK* genes belong to the serine/threonine kinase family. Based on protein motifs analysis, we found that all SmSnRK proteins had a serine/threonine kinase domain at the N-terminal, which further verified the accuracy of the *SmSnRK* family identification. Moreover, the SmSnRK1 and SmSnRK3 subfamilies contained unique domains at the C-terminal within their subfamily, as did SmSnRK2. This might due to the C-terminal of SmSnRK2 genes being divergent and it was not annotated in this study. Previous studies have shown that the exon–intron structure of the *SnRK2* subfamily in higher plants is conserved and most of the *SnRK2* genes have nine exons [60]. In *S. miltiorrhiza*, all *SmSnRK2* subfamily members had nine exons, in addition to *SmSnRK2.2* and *SmSnRK2.3*. Phylogenetic tree and exon–intron structure revealed that *SmSnRK2.2* and *SmSnRK2.3* were closely related and both had eight exons, indicating that the evolution and function of *SnRK2* might be relatively conserved. The *SmSnRK3* subfamily could be divided into an intron-rich group and an intron-poor group, indicating that the structural diversification of *SmSnRK3* may have been caused by gain or loss intron events, similarly to what has been suggested by results reported previously [61]. Previous studies found that the expression level of genes with fewer introns was higher than that of those with more introns, and the response to stress was more timely [62]. However, this was not observed for *SmSnRK* genes in this study.

*Cis*-regulatory elements, which were bound to transcriptional factors, played important roles in regulating gene expression. The *ABRE*, ABA-responsive element, has an ACGT core sequence and is recognized by bZIP transcriptional factors, which are phosphorylated by ABA-responsive SnRK2 kinases [63]. The promoter of *SmSnRKs* mainly contained light response elements and each promoter of *SmSnRK* had an average of 1.81 ABRE and 1.06 MeJA response motifs, suggesting that the expression of *SmSnRKs* was correlated with hormone signaling. The chromosomal location of *SmSnRK* genes showed that these genes were randomly distributed. The chromosomal location of tanshinone or salvianolic acid biosynthesis genes was also mapped to find out whether the *SmSnRK* genes clustered with these genes. *SmSnRK3.15* was clustered with *SmCPS1* and *SmKSL1*. The *SnRK2.2* and *SnRK2.3* in *S. miltiorrhiza* were clustered on chromosome LG8, and these protein sequences shared an identity of 91%, suggesting that these genes were included in tandem duplication occurrences.

Tanshinones and salvianolic acids were the main bioactive compounds of *S. miltiorrhiza*. Previous studies have shown that MeJA [46] and ABA [47] up-regulated the biosynthesis of these two compounds. Moreover, several genes involved in JA and ABA signaling pathways have been proven to promote the accumulation of tanshinones and salvianolic acids. For example, SmJAZ3 and SmJAZ9, the key repressors of JA signaling, negatively regulated the accumulation of tanshinones and salvianolic acids [64]. SmMYC2, the core component in JA signaling, up-regulated salvianolic acids accumulation [65]. SmSnRK2.6 (homology of OST), a key component in ABA signaling, interacted with SmAREB1 to promote the biosynthesis of salvianolic acids [49]. In *Artemisia annua*, AaAPK1 was an SnRK2 family member and responsive to ABA treatment, which acted as a positive regulator of artemisinin by phosphorylating AabZIP1 [66]. The expression of genes could give insight into the gene function. According to the transcriptome data for the *SnRK2* subfamily members, the transcripts of *SmSnRK2.1*, *SmSnRK2.2* and *SmSnRK2.6* were up-regulated by ABA treatment. However, the transcripts of other *SmSnRK2* members were slightly down-regulated by ABA treatment. In *A. thaliana*, SnRK2 could be divided into ABA-dependent and ABA-independent groups [67]. Moreover, Jia et al. reported that the overexpression of *SmSnRK2.6* enhanced the accumulation of salvianolic acids, whereas the overexpression of *SmSnRK2.3* did not [49]. Based on these findings, we suggested that *SmSnRK2* genes could also be divided into ABA-dependent and ABA-independent groups, and that *SmSnRK2* genes possessed functional diversity. The transcripts of *SmSnRK3.1*, *SmSnRK3.17* and *SmSnRK3.18* significantly increased and reached their highest at 0.5–1 h after ABA treatment, indicating that *SmSnRK3* genes might also be involved in ABA signaling. MeJA treatment decreased the transcripts of SmSnRK2.7, while other *SmSnRK1* and *SmSnRK2* genes remained almost unchanged. The transcripts of *SmSnRK3.18* significantly increased and reached their highest at 1 h after MeJA treatment, which was about 38 times higher than the control. The results showed that several *SmSnRK3* members might participate in ABA or MeJA signaling.

To further study the relationship between *SmSnRK* genes and tanshinone and salvianolic acid biosynthesis, a correlation analysis was conducted using the tissue expression analysis and hormone treatment of the *SmSnRK* gene with tanshinone and salvianolic acid biosynthesis genes. *SmSnRK3.15* and *SmSnRK3.18* were significantly related to the expression of the tanshinone biosynthesis gene; these genes were predominantly expressed in the roots of *S. miltiorrhiza*, indicating that *SmSnRK3.15* and *SmSnRK3.18* might regulate the expression of tanshinone biosynthesis. Based on correlation analysis, *SmSnRK3.10* and *SmSnRK3.12* were found to have a co-expression with salvianolic acid biosynthesis genes. The analysis revealed that most genes were involved in signal transduction biological processes, and we also found that SmSnRK2.2 interacted with and phosphorylated the SmAREB1 transcription factor, suggesting that several *SmSnRK* genes were important components in the signaling pathway for regulating tanshinone and salvianolic acid biosynthesis. More importantly, we identified that SmSnRK2.6 is a positive regulator in tanshinones biosynthesis, which was validated by our correlation analysis and shed lights on the role of the *SmSnRK* gene in tanshinones. This study provides comprehensive information about the *SmSnRK* family genes in *S. miltiorrhiza* and evidence for *SnRK* genes regulating plant metabolism.

## 4. Materials and Methods

### 4.1. Identification and Characterization of SmSnRK Genes in the S. miltiorrhiza Genome

To identify the *SmSnRK* family members in *S. miltiorrhiza*, the amino acid sequences of SnRK proteins from *A. thaliana* and *O. sativa* were downloaded from the TAIR database (http://www.arabidopsis.org, accessed on 15 May 2023) and Rice Annotation Project (RAP) (https://rapdb.dna.affrc.go.jp/, accessed on 15 May 2023). The protein and genome sequences of *S. miltiorrhiza* were downloaded from the Genome Warehouse (https://ngdc.cncb.ac.cn/, accessed on 15 May 2023) (PRJCA003150). In order to search all of the candidate *SmSnRK* genes, the Hidden Markov Model (HMM) profile of the Pkinase domain (PF00069) was obtained from the Pfam database (http://pfam.xfam.org/, accessed on 15 May 2023) [68] to find genes with a Pkinase domain by HMMER 3.0. Then, SnRK protein sequences from *A. thaliana* and *O. sativa* were set as the query and BLASTP was used against genome sequences of *S. miltiorrhiza*. Possible *SnRK* candidate genes were selected manually using the same genes which could be found in both HMM and BLASTP results. These non-redundant sequences were further verified by checking for the presence of the SnRK-related domains within the Pfam database, NCBI conserved domain database (https://www.ncbi.nlm.nih.gov/Structure/cdd/, accessed on 18 May 2023) [69] and SMART (http://smart.embl-heidelberg.de/, accessed on 18 May 2023) [70]. The properties of SnRK proteins, including the isoelectric point (pI), amino acids length and molecular weights (MW) were calculated with the ExPASy-Compute pI/Mw tool (https://www.expasy.org/resources/compute-pi-mw, accessed on 18 May 2023). The subcellular localization of the SmSnRK proteins was predicted with WoLP PSORT (https://wolfpsort.hgc.jp/, accessed on 18 May 2023).

### 4.2. Phylogenetic Analysis of SnRK Genes

A multiple sequence alignment of all of the SnRK members from *S. miltiorrhiza*, *A. thaliana* and *O. sativa* was conducted with ClustalW [71], using the default settings. A phylogenetic tree was built using the Neighbor-Joining (NJ) method [72] and bootstrap was set to 1000 with MEGA7.0 [73]. The Interactive Tree of Life (iTOL) website (https://itol.embl.de, accessed on 23 May 2023) was used to create the phylogenetic tree in this study.

### 4.3. Protein Motif and Gene Structural Analysis of SmSnRKs

The conserved motif analysis of the SmSnRK proteins was searched on the MEME website (https://meme-suite.org/meme/, accessed on 4 June 2023) [74] with the motif number of 15 and range from 6–50 amino acids residues. The gene structural analysis of the *SmSnRKs* was conducted with the Gene Structure Display Server (http://gsds.gao-lab.org/, accessed on 10 June 2023) [75].

### 4.4. Promoter Analysis and Chromosomal Location

The promoter sequences (2000 bp upstream from the initiation codon) of *SmSnRK* genes were extracted from the *S. miltiorrhiza* genome. Then, the promoter sequences were analyzed for *cis*-elements by the PlantCARE (http://bioinformatics.psb.ugent.be/webtools/plantcare/html/, accessed on 6 June 2023) website [76] and graphed by TBtools [77].

### 4.5. Expression Analysis of SmSnRK Genes

*S. miltiorrhiza* was cultivated in greenhouse at Zhejiang Chinese Medical University, Hangzhou. For tissue expression pattern analysis, different tissues including leaves, roots, stems and flowers of one-year-old *S. miltiorrhiza* plants were collected and frozen in liquid nitrogen immediately. After the samples were ground in liquid nitrogen, the RNApure Plant Kit (Tiangen, Beijing, China) was used for the RNA extraction of samples. For qRT-PCR analysis, cDNA was synthesized by the reverse transcription method (Vazyme). qRT-PCR was conducted on a StepOne Plus Real-Time PCR system (Applied Biosystems, Waltham, MA, USA) and ChamQ Universal SYBR qPCR Master Mix (Vazyme, Nanjing, China) was used. The PCR program was followed with the protocol. The relative expression was calculated by the 2^−ΔΔCt^ algorithm. *SmActin* was used as an internal reference gene [78]. Primers used in this study are listed in Supplementary Materials Table S2. Triple biological repeats (each replicate containing five plants) were performed for each sample, and each reaction was measured in triplicate.

To study the expression pattern of *SmSnRK* genes responses to hormone, the transcriptome data of *S. miltiorrhiza* treated with ABA [79] or MeJA [80] were obtained from previous studies. The FPKM of *SmSnRKs* was used to analyze the expression of *SmSnRK* genes and the FPKM value was standardized by log_2_ for heatmap presentation. The heatmap was based on the transcriptome data of hormone treatment and drawn by the TBtools [77].

### 4.6. Gene Ontology and Correlation Analysis

The Gene Ontology information, including the biological processes and molecular function of *SmSnRKs*, was analyzed on the PANTHER database (https://ngdc.cncb.ac.cn/databasecommons/, accessed on 23 September 2023) [81]. The correlation analysis of SmSnRKs and tanshinones biosynthesis genes was based on the Pearson correlation coefficient value with R > 0.8 and the *p*-value < 0.05. The relevance between *SmSnRKs* and tanshinone biosynthesis genes was drawn by Cytoscape_v3.7.2 software [82].

### 4.7. Yeast Two-Hybrid (Y2H) Assays

The Y2H assays were performed as described previously [83]. The coding region of *SmSnRK2.2* and *SmAREB1* were ligated into pGBKT7 and pGADT7 constructing the BD-*SmSnRK2.2* and AD-*SmAREB1*, respectively. BD-*SmSnRK2.2* and AD-*SmAREB1* were co-transformed into yeast competent cells and the interaction was tested on selective medium (SD/-Trp-Leu and SD/-Leu-Trp-His-Ade). The combination of pGBKT7/AD-*SmAREB1*, pGADT7/BD-*SmSnRK2.2* and pGBKT7/pGADT7 served as the negative control. Primers used in this study are listed in Supplementary Materials Table S2.

### 4.8. Bimolecular Fluorescence Complementation (BiFc) Analysis

The BiFc assays were performed as described previously [83]. The CDS sequence of *SmSnRK2.2* was amplified and ligated into a pXY104 vector to generate the pXY104-*SmSnRK2.2*-cYFP construct. The CDS sequences of *SmAREB1* were amplified and ligated into the vector pXY106 to generate the pXY106-nYFP-*SmAREB1* construct, respectively. Then, these vectors were individually transformed into *Agrobacterium tumefaciens* GV3101, and co-infiltrated into tobacco leaves. After 48 h of infiltration, the fluorescence was captured by a confocal laser-scanning microscope (Zeiss LSM-710, Carl Zeiss, Shanghai, China). The combination of pXY104-*SmSnRK2.2*-cYFP/pXY106 and pXY106-nYFP-*SmAREB1*/pXY104 served as the negative control. Primers used in this study are listed in Supplementary Materials Table S2.

### 4.9. Phosphorylation Assays

For phosphorylation assays, the SmSnRK2.2 and SmAREB1 proteins were obtained by prokaryotic protein expression. First, the coding sequences of *SmSnRK2.2* and *SmAREB1* were inserted into the pGEX-4T-1 and pET32a vectors, respectively. Then, the vectors were transformed into *E. coli* BL21 (DE3) for protein production. The purified proteins of SmSnRK2.2 and SmAREB1 were co-incubated in kinase buffer (20 mM Tris-HCl pH 7.5, 20 mM MgCl_2_, 1 mM DTT and 0.5 mM ATP) for 1 h, separated by SDS-PAGE with phos-tag (Wako) of 25 μM and 50 μM MnCl_2_ and subjected to immunoblot analysis with an anti-GST antibody (Transgen, Beijing, China). Primers used in this study are listed in Supplementary Materials Table S2.

### 4.10. Trangenic Method

In order to generate the RNAi-*SmSnRK2.6* construct, a 250 bp specific coding sequence of *SmSnRK2.6* was inserted into the pFGC5941 vector between *Nco*I and *Asc*I restriction sites. After that, the specific fragment was then reversibly inserted into the *Bam*HI and *Xba*I restriction sites of pFGC5941, which already contained the specific coding sequence. Then, the construct was transformed into *A. tumefaciens* strain C58C1 and transgenic hairy roots production was based on our previous study [83]. Primers used in this study are listed in Supplementary Materials Table S2.

## Data Availability

The data presented in this study are available in Supplementary Materials.

## Supplementary Materials

The following supporting information can be downloaded at: https://www.mdpi.com/article/10.3390/plants13070994/s1, Figure S1: Motifs and gene structure of *SmSnRK*; Figure S2: Chromosomal location of *SmSnRK*; Figure S3: RNA-seq expression analysis of *SmSnRK* genes under MeJA treatment; Figure S4: RNA-seq expression analysis of *SmSnRK* genes under ABA treatment; Figure S5: *SmSnRK2.6* expression levels in RNAi lines; Table S1: Physicochemical properties of SmSnRK; Table S2: Primers used in this study; Table S3: SmSnRK sequences.

## References

[1] Zhang H., Zhu J., Gong Z., Zhu J.K. (2022). Abiotic stress responses in plants. Nat. Rev. Genet..

[2] Chen X., Ding Y., Yang Y., Song C., Wang B., Yang S., Guo Y., Gong Z. (2021). Protein kinases in plant responses to drought, salt, and cold stress. J. Integr. Plant Biol..

[3] Hrabak E.M., Chan C.W., Gribskov M., Harper J.F., Choi J.H., Halford N., Kudla J., Luan S., Nimmo H.G., Sussman M.R. (2003). The *Arabidopsis* CDPK-SnRK superfamily of protein kinases. Plant Physiol..

[4] Coello P., Hey S.J., Halford N.G. (2011). The sucrose non-fermenting-1-related (SnRK) family of protein kinases: Potential for manipulation to improve stress tolerance and increase yield. J. Exp. Bot..

[5] Belda-Palazón B., Adamo M., Valerio C., Ferreira L.J., Confraria A., Reis-Barata D., Rodrigues A., Meyer C., Rodriguez P.L., Baena-González E. (2020). A dual function of SnRK2 kinases in the regulation of SnRK1 and plant growth. Nat. Plants.

[6] Baena-González E., Rolland F., Thevelein J.M., Sheen J. (2007). A central integrator of transcription networks in plant stress and energy signalling. Nature.

[7] Margalha L., Valerio C., Baena-González E. (2016). Plant SnRK1 Kinases: Structure, Regulation, and Function. Exp. Suppl..

[8] Baena-González E., Sheen J. (2008). Convergent energy and stress signaling. Trends Plant Sci..

[9] Nukarinen E., Nägele T., Pedrotti L., Wurzinger B., Mair A., Landgraf R., Börnke F., Hanson J., Teige M., Baena-Gonzalez E. (2016). Quantitative phosphoproteomics reveals the role of the AMPK plant ortholog SnRK1 as a metabolic master regulator under energy deprivation. Sci. Rep..

[10] Ramon M., Dang T.V.T., Broeckx T., Hulsmans S., Crepin N., Sheen J., Rolland F. (2019). Default Activation and Nuclear Translocation of the Plant Cellular Energy Sensor SnRK1 Regulate Metabolic Stress Responses and Development. Plant Cell.

[11] Boudsocq M., Barbier-Brygoo H., Laurière C. (2004). Identification of nine sucrose nonfermenting 1-related protein kinases 2 activated by hyperosmotic and saline stresses in *Arabidopsis thaliana*. J. Biol. Chem..

[12] Kobayashi Y., Yamamoto S., Minami H., Kagaya Y., Hattori T. (2004). Differential activation of the rice sucrose nonfermenting1-related protein kinase2 family by hyperosmotic stress and abscisic acid. Plant Cell.

[13] Kulik A., Wawer I., Krzywińska E., Bucholc M., Dobrowolska G. (2011). SnRK2 protein kinases—Key regulators of plant response to abiotic stresses. OMICS.

[14] Kobayashi Y., Murata M., Minami H., Yamamoto S., Kagaya Y., Hobo T., Yamamoto A., Hattori T. (2005). Abscisic acid-activated SNRK2 protein kinases function in the gene-regulation pathway of ABA signal transduction by phosphorylating ABA response element-binding factors. Plant J..

[15] Furihata T., Maruyama K., Fujita Y., Umezawa T., Yoshida R., Shinozaki K., Yamaguchi-Shinozaki K. (2006). Abscisic acid-dependent multisite phosphorylation regulates the activity of a transcription activator AREB1. Proc. Natl. Acad. Sci. USA.

[16] Fujii H., Verslues P.E., Zhu J.K. (2007). Identification of two protein kinases required for abscisic acid regulation of seed germination, root growth, and gene expression in *Arabidopsis*. Plant Cell.

[17] Miyazono K., Miyakawa T., Sawano Y., Kubota K., Kang H.J., Asano A., Miyauchi Y., Takahashi M., Zhi Y., Fujita Y. (2009). Structural basis of abscisic acid signalling. Nature.

[18] Geiger D., Scherzer S., Mumm P., Stange A., Marten I., Bauer H., Ache P., Matschi S., Liese A., Al-Rasheid K.A. (2009). Activity of guard cell anion channel SLAC1 is controlled by drought-stress signaling kinase-phosphatase pair. Proc. Natl. Acad. Sci. USA.

[19] Sato A., Sato Y., Fukao Y., Fujiwara M., Umezawa T., Shinozaki K., Hibi T., Taniguchi M., Miyake H., Goto D.B. (2009). Threonine at position 306 of the KAT1 potassium channel is essential for channel activity and is a target site for ABA-activated SnRK2/OST1/SnRK2.6 protein kinase. Biochem. J..

[20] Sirichandra C., Gu D., Hu H.C., Davanture M., Lee S., Djaoui M., Valot B., Zivy M., Leung J., Merlot S. (2009). Phosphorylation of the Arabidopsis AtrbohF NADPH oxidase by OST1 protein kinase. FEBS Lett..

[21] Nakashima K., Fujita Y., Kanamori N., Katagiri T., Umezawa T., Kidokoro S., Maruyama K., Yoshida T., Ishiyama K., Kobayashi M. (2009). Three Arabidopsis SnRK2 protein kinases, SRK2D/SnRK2.2, SRK2E/SnRK2.6/OST1 and SRK2I/SnRK2.3, involved in ABA signaling are essential for the control of seed development and dormancy. Plant Cell Physiol..

[22] Rachowka J., Anielska-Mazur A., Bucholc M., Stephenson K., Kulik A. (2023). SnRK2.10 kinase differentially modulates expression of hub WRKY transcription factors genes under salinity and oxidative stress in *Arabidopsis thaliana*. Front. Plant Sci..

[23] Soma F., Takahashi F., Suzuki T., Shinozaki K., Yamaguchi-Shinozaki K. (2020). Plant Raf-like kinases regulate the mRNA population upstream of ABA-unresponsive SnRK2 kinases under drought stress. Nat. Commun..

[24] Kolukisaoglu U., Weinl S., Blazevic D., Batistic O., Kudla J. (2004). Calcium sensors and their interacting protein kinases: Genomics of the *Arabidopsis* and rice CBL-CIPK signaling networks. Plant Physiol..

[25] Kimura S., Kawarazaki T., Nibori H., Michikawa M., Imai A., Kaya H., Kuchitsu K. (2013). The CBL-interacting protein kinase CIPK26 is a novel interactor of *Arabidopsis* NADPH oxidase AtRbohF that negatively modulates its ROS-producing activity in a heterologous expression system. J. Biochem..

[26] Luan S., Lan W., Chul Lee S. (2009). Potassium nutrition, sodium toxicity, and calcium signaling: Connections through the CBL-CIPK network. Curr. Opin. Plant Biol..

[27] Batistic O., Kudla J. (2004). Integration and channeling of calcium signaling through the CBL calcium sensor/CIPK protein kinase network. Planta.

[28] Qiu Q.S., Guo Y., Dietrich M.A., Schumaker K.S., Zhu J.K. (2002). Regulation of SOS1, a plasma membrane Na^+^/H^+^ exchanger in *Arabidopsis thaliana*, by SOS_2_ and SOS_3_. Proc. Natl. Acad. Sci. USA.

[29] Liu J., Ishitani M., Halfter U., Kim C.S., Zhu J.K. (2000). The *Arabidopsis thaliana* SOS_2_ gene encodes a protein kinase that is required for salt tolerance. Proc. Natl. Acad. Sci. USA.

[30] Halfter U., Ishitani M., Zhu J.K. (2000). The Arabidopsis SOS_2_ protein kinase physically interacts with and is activated by the calcium-binding protein SOS3. Proc. Natl. Acad. Sci. USA.

[31] Guo Y., Xiong L., Song C.P., Gong D., Halfter U., Zhu J.K. (2002). A calcium sensor and its interacting protein kinase are global regulators of abscisic acid signaling in *Arabidopsis*. Dev. Cell.

[32] D’Angelo C., Weinl S., Batistic O., Pandey G.K., Cheong Y.H., Schültke S., Albrecht V., Ehlert B., Schulz B., Harter K. (2006). Alternative complex formation of the Ca-regulated protein kinase CIPK1 controls abscisic acid-dependent and independent stress responses in *Arabidopsis*. Plant J..

[33] Cheong Y., Pandey G.K., Grant J.J., Batistic O., Li L., Kim B.G., Lee S.C., Kudla J., Luan S. (2007). Two calcineurin B-like calcium sensors, interacting with protein kinase CIPK23, regulate leaf transpiration and root potassium uptake in *Arabidopsis*. Plant J..

[34] Zhou X., Hao H., Zhang Y., Bai Y., Zhu W., Qin Y., Yuan F., Zhao F., Wang M., Hu J. (2015). SOS_2_-LIKE PROTEIN KINASE5, an SNF1-RELATED PROTEIN KINASE3-Type Protein Kinase, Is Important for Abscisic Acid Responses in *Arabidopsis* through Phosphorylation of ABSCISIC ACID-INSENSITIVE5. Plant Physiol..

[35] Wang L., Ma R., Liu C., Liu H., Zhu R., Guo S., Tang M., Li Y., Niu J., Fu M. (2017). *Salvia miltiorrhiza*: A Potential Red Light to the Development of Cardiovascular Diseases. Curr. Pharm. Des..

[36] Sun L., Zhang Y.N. (2022). Compound Danshen dripping pills in treating with coronary heart disease: A protocol for systematic review and meta-analysis. Medicine.

[37] Xu H., Song J., Luo H., Zhang Y., Li Q., Zhu Y., Xu J., Li Y., Song C., Wang B. (2016). Analysis of the Genome Sequence of the Medicinal Plant *Salvia miltiorrhiza*. Mol. Plant.

[38] Song Z., Lin C., Xing P., Fen Y., Jin H., Zhou C., Gu Y.Q., Wang J., Li X. (2020). A high-quality reference genome sequence of *Salvia miltiorrhiza* provides insights into tanshinone synthesis in its red rhizomes. Plant Genome.

[39] Ma Y., Cui G., Chen T., Ma X., Wang R., Jin B., Yang J., Kang L., Tang J., Lai C. (2021). Expansion within the CYP71D subfamily drives the heterocyclization of tanshinones synthesis in *Salvia miltiorrhiza*. Nat. Commun..

[40] Li C., Li D., Shao F., Lu S. (2015). Molecular cloning and expression analysis of *WRKY* transcription factor genes in *Salvia miltiorrhiza*. BMC Genom..

[41] Zhang X., Luo H., Xu Z., Zhu Y., Ji A., Song J., Chen S. (2015). Genome-wide characterisation and analysis of *bHLH* transcription factors related to tanshinone biosynthesis in *Salvia miltiorrhiza*. Sci. Rep..

[42] Li L., Liu Y., Huang Y., Li B., Ma W., Wang D., Cao X., Wang Z. (2021). Genome-Wide Identification of the *TIFY* Family in *Salvia miltiorrhiza* Reveals That SmJAZ3 Interacts with SmWD40-170, a Relevant Protein That Modulates Secondary Metabolism and Development. Front. Plant Sci..

[43] Li Q., Feng J., Chen L., Xu Z., Zhu Y., Wang Y., Xiao Y., Chen J., Zhou Y., Tan H. (2019). Genome-Wide Identification and Characterization of *Salvia miltiorrhiza* Laccases Reveal Potential Targets for Salvianolic Acid B Biosynthesis. Front. Plant Sci..

[44] Li Y., Tong Y., Ye J., Zhang C., Li B., Hu S., Xue X., Tian Q., Wang Y., Li L. (2023). Genome-Wide Characterization of *B-Box*. Gene Family in *Salvia miltiorrhiza*. Int. J. Mol. Sci..

[45] Chai S., Li K., Deng X., Wang L., Jiang Y., Liao J., Yang R., Zhang L. (2023). Genome-Wide Analysis of the *MADS-box* Gene Family and Expression Analysis during Anther Development in *Salvia miltiorrhiza*. Int. J. Mol. Sci..

[46] Wang X., Cui G., Huang L., Qiu D. (2007). Effects of methyl jasmonat on accumulation and release of tanshinones in suspension cultures of *Salvia miltiorrhiza* hairy root. China J. Chin. Mater. Med..

[47] Liang Z., Ma Y., Xu T., Cui B., Liu Y., Guo Z., Yang D. (2013). Effects of abscisic acid, gibberellin, ethylene and their interactions on production of phenolic acids in *Salvia miltiorrhiza* bunge hairy roots. PLoS ONE.

[48] Mason H.S., DeWald D.B., Mullet J.E. (1993). Identification of a methyl jasmonate-responsive domain in the soybean vspB promoter. Plant Cell.

[49] Jia Y., Bai Z., Pei T., Ding K., Liang Z., Gong Y. (2017). The Protein Kinase SmSnRK2.6 Positively Regulates Phenolic Acid Biosynthesis in *Salvia miltiorrhiza* by Interacting with SmAREB1. Front. Plant Sci..

[50] Li Q., Zhao H., Wang X., Kang J., Lv B., Dong Q., Li C., Chen H., Wu Q. (2020). Tartary Buckwheat Transcription Factor FtbZIP5, Regulated by FtSnRK2.6, Can Improve Salt/Drought Resistance in Transgenic *Arabidopsis*. Int. J. Mol. Sci..

[51] Ma X., Li Q.H., Yu Y.N., Qiao Y.M., Haq S.U., Gong Z.H. (2020). The CBL-CIPK Pathway in Plant Response to Stress Signals. Int. J. Mol. Sci..

[52] Li R., Radani Y., Ahmad B., Movahedi A., Yang L. (2022). Identification and characteristics of *SnRK* genes and cold stress-induced expression profiles in *Liriodendron chinense*. BMC Genom..

[53] Zhu W., Wu D., Jiang L., Ye L. (2020). Genome-wide identification and characterization of *SnRK* family genes in *Brassica napus*. BMC Plant Biol..

[54] Ai D., Wang Y., Wei Y., Zhang J., Meng J., Zhang Y. (2022). Comprehensive identification and expression analyses of the *SnRK* gene family in *Casuarina equisetifolia* in response to salt stress. BMC Plant Biol..

[55] Wang L., Hu W., Sun J., Liang X., Yang X., Wei S., Wang X., Zhou Y., Xiao Q., Yang G. (2015). Genome-wide analysis of *SnRK* gene family in *Brachypodium distachyon* and functional characterization of *BdSnRK2.9*. Plant Sci..

[56] Liu Z., Ge X., Yang Z., Zhang C., Zhao G., Chen E., Liu J., Zhang X., Li F. (2017). Genome-wide identification and characterization of *SnRK2* gene family in cotton (*Gossypium hirsutum* L.). BMC Genet..

[57] Mishra S., Sharma P., Singh R., Tiwari R., Singh G.P. (2021). Genome-wide identification and expression analysis of sucrose nonfermenting-1-related protein kinase (*SnRK*) genes in *Triticum aestivum* in response to abiotic stress. Sci. Rep..

[58] Cervera-Torres C., Arthikala M.K., Lara M., Blanco L., Nanjareddy K. (2022). Comprehensive Analysis of *Phaseolus vulgaris SnRK* Gene Family and Their Expression during Rhizobial and Mycorrhizal Symbiosis. Genes.

[59] Shi M., Du Z.Y., Hua Q., Kai G. (2021). CRISPR/Cas9-mediated targeted mutagenesis of bZIP2 in *Salvia miltiorrhiza* leads to promoted phenolic acid biosynthesis. Ind. Crops Prod..

[60] Saha J., Chatterjee C., Sengupta A., Gupta K., Gupta B. (2014). Genome-wide analysis and evolutionary study of sucrose non-fermenting 1-related protein kinase 2 (*SnRK2*) gene family members in *Arabidopsis* and *Oryza*. Comput. Biol. Chem..

[61] Zhu K., Chen F., Liu J., Chen X., Hewezi T., Cheng Z.M. (2016). Evolution of an intron-poor cluster of the CIPK gene family and expression in response to drought stress in soybean. Sci. Rep..

[62] Jeffares D.C., Penkett C.J., Bähler J. (2008). Rapidly regulated genes are intron poor. Trends Genet..

[63] Sun X., Li Y., Cai H., Bai X., Wei J., Li Z., Zhu Y. (2011). *Arabidopsis* bZIP1 Transcription Factor Binding to ABRE cis-Element Regulates Abscisic Acid Signal Transduction. Zuo Wu Xue Bao.

[64] Shi M., Zhou W., Zhang J., Huang S., Wang H., Kai G. (2016). Methyl jasmonate induction of tanshinone biosynthesis in *Salvia miltiorrhiza* hairy roots is mediated by JASMONATE ZIM-DOMAIN repressor proteins. Sci. Rep..

[65] Yang N., Zhou W., Su J., Wang X., Li L., Wang L., Cao X., Wang Z. (2017). Overexpression of *SmMYC2* Increases the Production of Phenolic Acids in *Salvia miltiorrhiza*. Front. Plant Sci..

[66] Zhang F., Xiang L., Yu Q., Zhang H., Zhang T., Zeng J., Geng C., Li L., Fu X., Shen Q. (2018). ARTEMISININ BIOSYNTHESIS PROMOTING KINASE 1 positively regulates artemisinin biosynthesis through phosphorylating AabZIP1. J. Exp. Bot..

[67] Yoshida T., Mogami J., Yamaguchi-Shinozaki K. (2014). ABA-dependent and ABA-independent signaling in response to osmotic stress in plants. Curr. Opin. Plant Biol..

[68] Finn R.D., Coggill P., Eberhardt R.Y., Eddy S.R., Mistry J., Mitchell A.L., Potter S.C., Punta M., Qureshi M., Sangrador-Vegas A. (2016). The Pfam protein families database: Towards a more sustainable future. Nucleic Acids Res..

[69] Marchler-Bauer A., Lu S., Anderson J.B., Chitsaz F., Derbyshire M.K., DeWeese-Scott C., Fong J.H., Geer L.Y., Geer R.C., Gonzales N.R. (2011). CDD: A Conserved Domain Database for the functional annotation of proteins. Nucleic Acids Res..

[70] Letunic I., Doerks T., Bork P. (2012). SMART 7: Recent updates to the protein domain annotation resource. Nucleic Acids Res..

[71] Larkin M.A., Blackshields G., Brown N.P., Chenna R., McGettigan P.A., McWilliam H., Valentin F., Wallace I.M., Wilm A., Lopez R. (2007). Clustal W and Clustal X version 2.0. Bioinformatics.

[72] Saitou N., Nei M. (1987). The neighbor-joining method: A new method for reconstructing phylogenetic trees. Mol. Biol. Evol..

[73] Kumar S., Stecher G., Tamura K. (2016). MEGA7: Molecular Evolutionary Genetics Analysis Version 7.0 for Bigger Datasets. Mol. Biol. Evol..

[74] Bailey T.L., Boden M., Buske F.A., Frith M., Grant C.E., Clementi L., Ren J., Li W.W., Noble W.S. (2009). MEME SUITE: Tools for motif discovery and searching. Nucleic Acids Res..

[75] Hu B., Jin J., Guo A.Y., Zhang H., Luo J., Gao G. (2015). GSDS 2.0: An upgraded gene feature visualization server. Bioinformatics.

[76] Lescot M., Déhais P., Thijs G., Marchal K., Moreau Y., Van de Peer Y., Rouzé P., Rombauts S. (2002). PlantCARE, a database of plant cis-acting regulatory elements and a portal to tools for in silico analysis of promoter sequences. Nucleic Acids Res..

[77] Chen C., Chen H., Zhang Y., Thomas H.R., Frank M.H., He Y., Xia R. (2020). TBtools: An Integrative Toolkit Developed for Interactive Analyses of Big Biological Data. Mol. Plant.

[78] Deng C., Hao X., Shi M., Fu R., Wang Y., Zhang Y., Zhou W., Feng Y., Makunga N.P., Kai G. (2019). Tanshinone production could be increased by the expression of *SmWRKY2* in *Salvia miltiorrhiza* hairy roots. Plant Sci..

[79] Deng C., Shi M., Fu R., Zhang Y., Wang Q., Zhou Y., Wang Y., Ma X., Kai G. (2020). ABA-responsive transcription factor bZIP1 is involved in modulating biosynthesis of phenolic acids and tanshinones in *Salvia miltiorrhiza*. J. Exp. Bot..

[80] Zhou W., Huang Q., Wu X., Zhou Z., Ding M., Shi M., Huang F., Li S., Wang Y., Kai G. (2017). Comprehensive transcriptome profiling of *Salvia miltiorrhiza* for discovery of genes associated with the biosynthesis of tanshinones and phenolic acids. Sci. Rep..

[81] Mi H., Lazareva-Ulitsky B., Loo R., Kejariwal A., Vandergriff J., Rabkin S., Guo N., Muruganujan A., Doremieux O., Campbell M.J. (2005). The PANTHER database of protein families, subfamilies, functions and pathways. Nucleic Acids Res..

[82] Shannon P., Markiel A., Ozier O., Baliga N.S., Wang J.T., Ramage D., Amin N., Schwikowski B., Ideker T. (2003). Cytoscape: A software environment for integrated models of biomolecular interaction networks. Genome Res..

[83] Liu S., Gao X., Shi M., Sun M., Li K., Cai Y., Chen C., Wang C., Maoz I., Guo X. (2023). Jasmonic acid regulates the biosynthesis of medicinal metabolites via the JAZ9-MYB76 complex in *Salvia miltiorrhiza*. Hortic. Res..

