# Supplementary figures and images for: Genome-Wide Identification and Expression Analysis of Sucrose Nonfermenting 1-Related Protein Kinase (SnRK) Genes in Salvia miltiorrhiza in Response to Hormone
Source: Plants (Basel). 2024 Mar 30;13(7):994. doi: 10.3390/plants13070994 (PMC11013873; doi:10.3390/plants13070994)

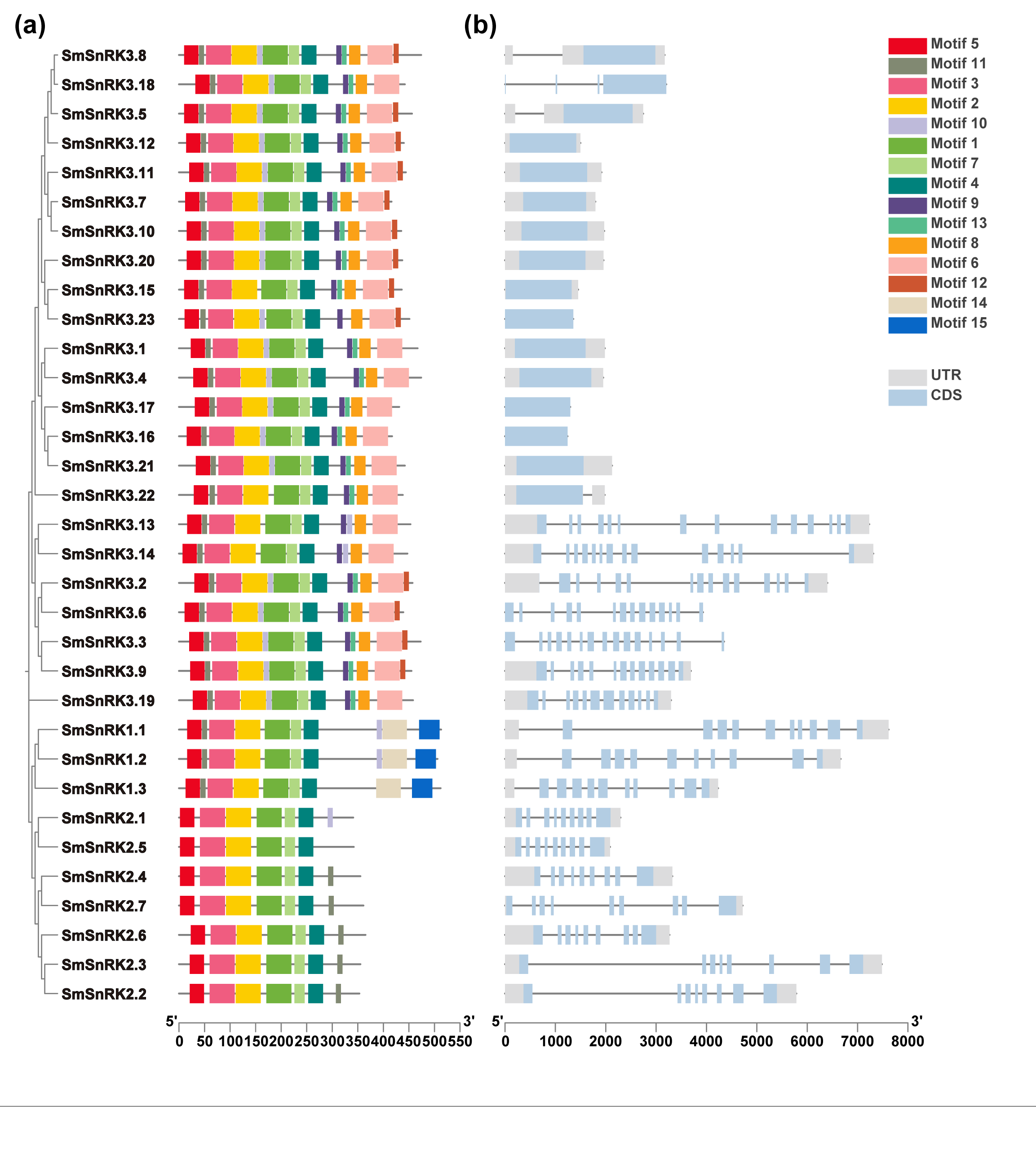

Supplement: Supplementary file 1 [file plants-13-00994-s001.zip › Figure S1.tif]

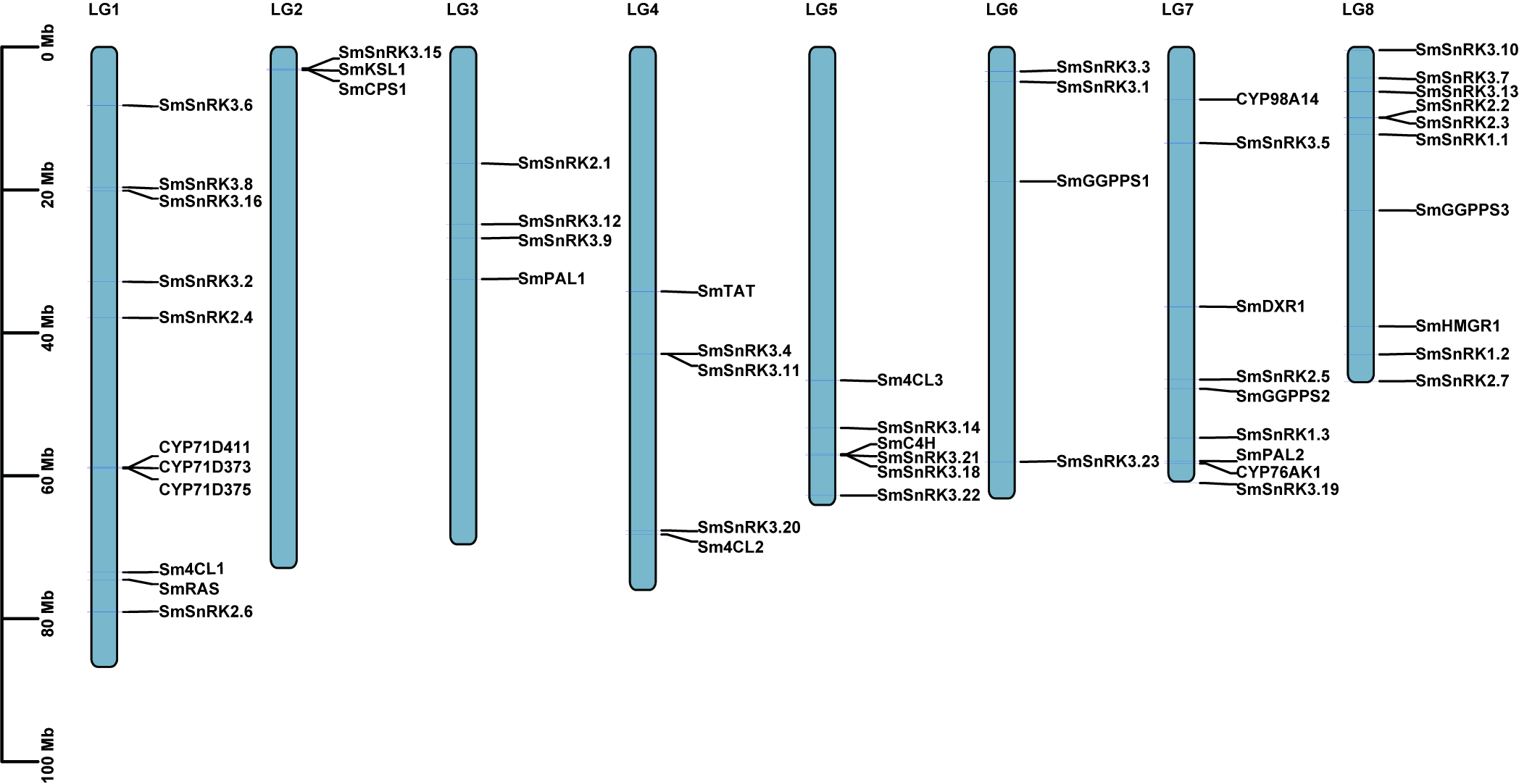

Supplement: Supplementary file 1 [file plants-13-00994-s001.zip › Figure S2.tif]

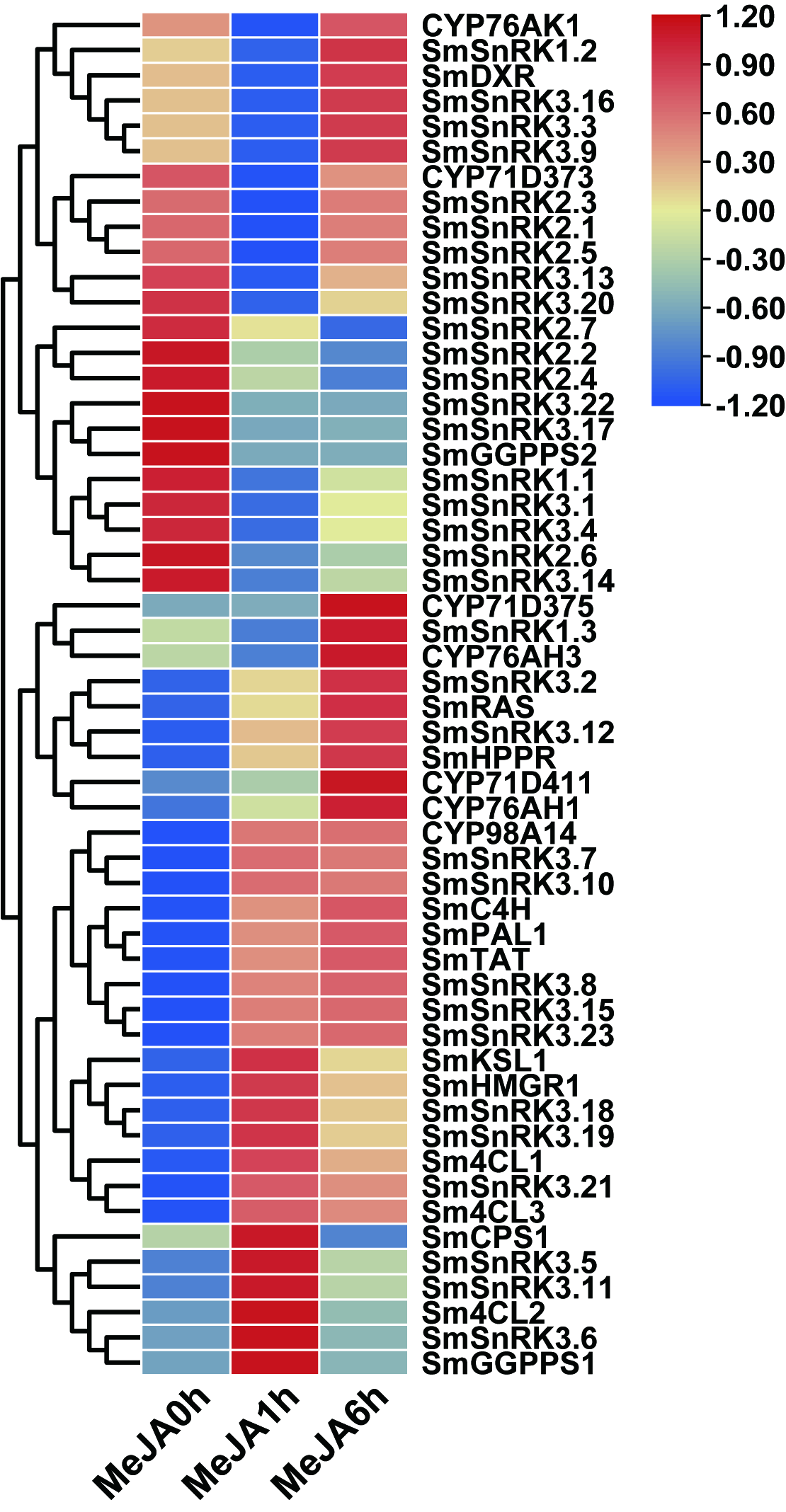

Supplement: Supplementary file 1 [file plants-13-00994-s001.zip › Figure S3.tif]

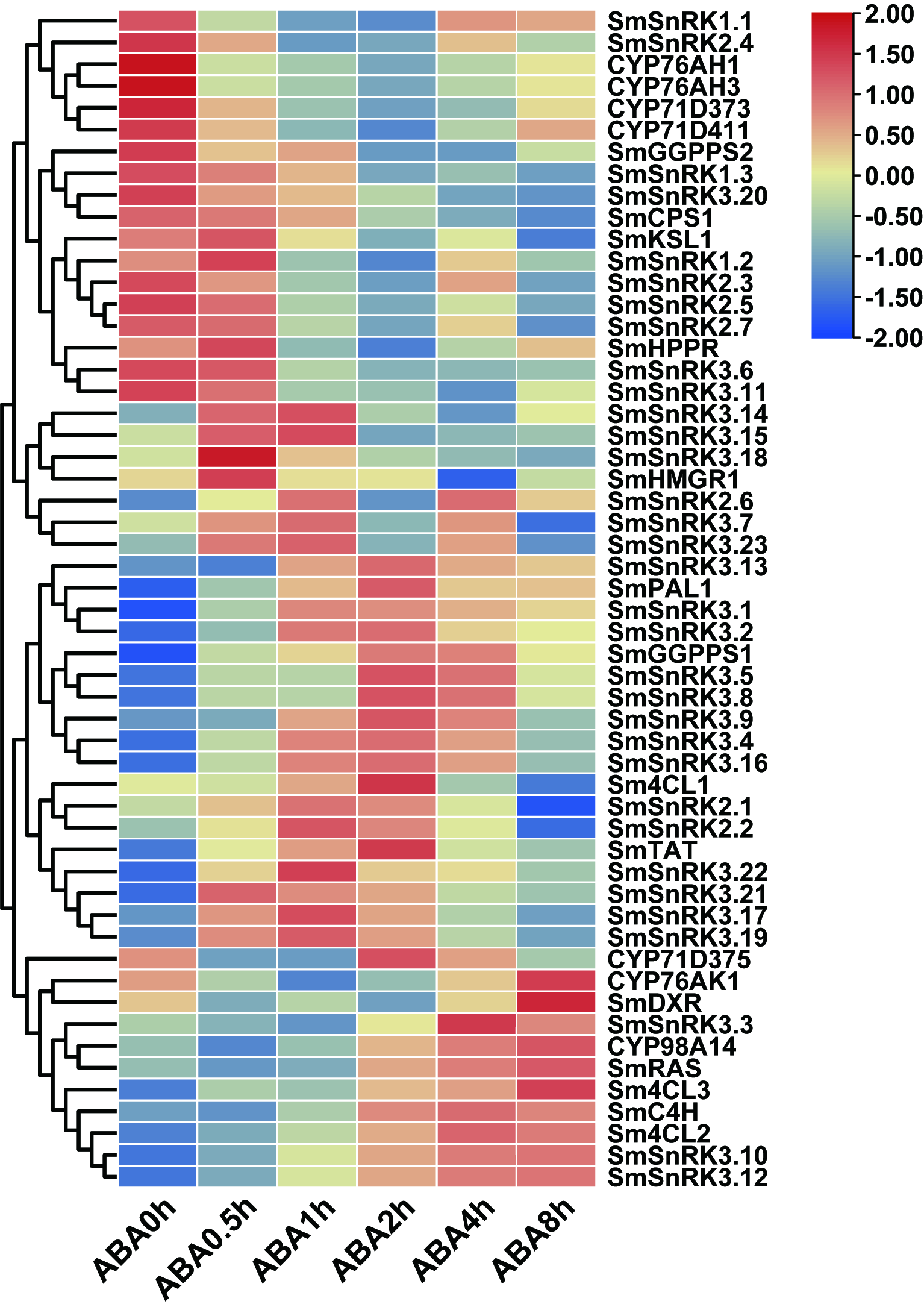

Supplement: Supplementary file 1 [file plants-13-00994-s001.zip › Figure S4.tif]

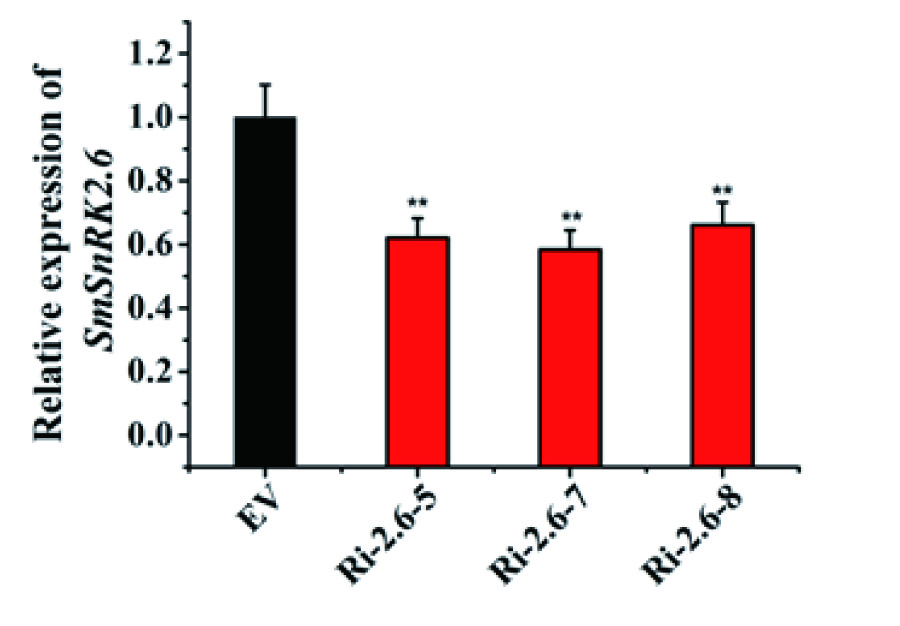

Supplement: Supplementary file 1 [file plants-13-00994-s001.zip › Figure S5.jpg]
